# Supplementary material for: A Critical Analysis of Indigenous Systems and Practices of Solid Waste Management in Rural Communities: The Case of Maseru in Lesotho
Source: Int J Environ Res Public Health. 2022 Sep 15;19(18):11654. doi: 10.3390/ijerph191811654 (PMC9517233; doi:10.3390/ijerph191811654)
Supplement: Supplementary file 1 [file ijerph-19-11654-s001.zip › ijerph-1856559-supplementary.pdf]

Supplementary file S1

FACULTY OF HEALTH SCIENCES
RESEARCH ETHICS COMMITTEE

NHREC Registration: REC 241112-035

ETHICAL CLEARANCE LETTER
(RECX 2.0)

Student/Researcher Name	Flory Senekane	Student Number	201332370
Supervisor Name	Makhene, Agnes		
Department	Environmental Health		
Research Title	A CRITICAL ANALYSIS OF THE INDIGENOUS SYSTEMS AND PRACTICES OF SOLID WASTE MANAGEMENT IN RURAL COMMUNITIES OF MASERU IN LESOT AND THE IMPACT ON THE ENVIRONMENT AND HUMAN WELLBEING TOWARDS IDENTIFYING APPROPRIATE CONTROL MEASURES		
Date	26 November 2020	Clearance Number	**REC-819-2020**

Approval of the research proposal with details given above is granted, subject to any conditions under 1 below, and is [illegible] until 2021/11/25.

1. Conditions:
Gatekeeper permission, as required.

2. Renewal:
It is required that this ethical clearance is renewed annually, within two weeks of the date indicated above. Renewal mu[illegible] done using the Ethical Clearance Renewal Form (REC 10.0), to be completed and submitted to the Faculty Administrat[illegible] office. *See Section 12 of the REC Standard Operating Procedures.*

3. Amendments:
Any envisaged amendments to the research proposal that has been granted ethical clearance must be submitted to the REC using the Research Proposal Amendment Application Form (REC 8.0) <u>prior to</u> the research being amended. Amendments to research may only be carried out once a new ethical clearance letter is issued. *See Section 13 of the [illegible] Standard Operating Procedures.*

4. Adverse Events, Deviations or Non-compliance:
Adverse events, research proposal deviations or non-compliance <u>must be reported</u> within the stipulated time-frames us[illegible] the Adverse Event Reporting Form (REC 9.0). *See Section 14 of the REC Standard Operating Procedures.*

The REC wishes you all the best for your studies.

Yours sincerely,

Prof. Christopher Stein

Supplementary file S2

TEL: +266 58700811
+266 27008007

OFISING EA MORENA OA SEHLOOHO
MATSIENG
BOX 1
MATSIENG 192

MATS/EDC/7
SBS/MER

La 6 'Mesa 2021

Marena,

Ke hlahisa kapele ho liofisi tsa lona 'M'e Mpinane Senekane ea kopang tumello ea ho etsa boithuto mabapi le mekhoa eo sechaba se boliseng ba lona se e sebelisang ho boloka bohloeki tikolohong eo ba phelang ho eona. 'M'e Mpinane Senekane ke moithuti oa University of Johannesburg.

Le kupuoa ke hona ho amohela 'M'e Mpinane Senekane kahara metse ea lona esita le ho molumella ho buisana le sechaba se boliseng ba lona ho tloha ka 'Mesa 2021.

MORENA OA SEHLOOHO
MATSIENG
0[illegible] -04- 2021
BOX 1 • MATSIENG
MASERU LESOTHO

Ka Litumeliso
'Na

Act. Morena oa Sehlooho Matsieng

Supplementary file S3

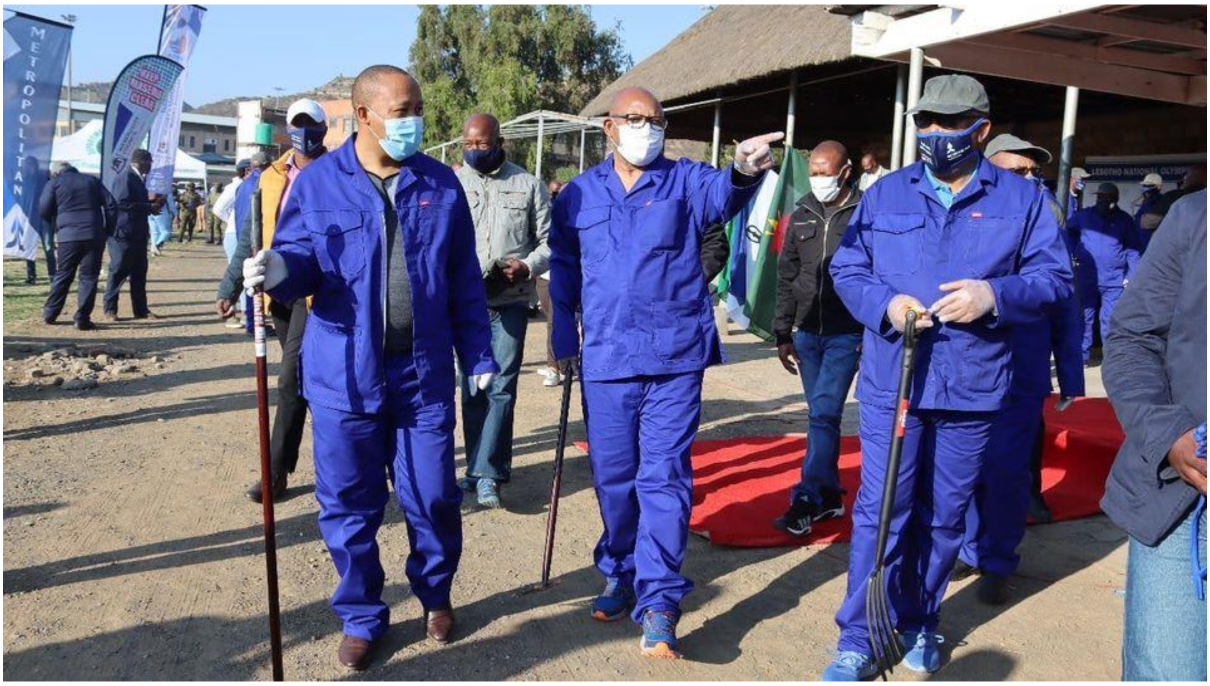

The Prime Minister, Dr Moeketsi Majoro on Friday launched the ‘Operation Hloekisa Lesotho’ cleaning campaign in Maseru.
